# Supplementary material for: Spatially explicit models of density improve estimates of Eastern Bering Sea beluga (Delphinapterus leucas) abundance and distribution from line-transect surveys
Source: PeerJ. 2025 Sep 30;13:e20077. doi: 10.7717/peerj.20077 (PMC13353160; doi:10.7717/peerj.20077)
Supplement: Supplemental Information 1 [file peerj-13-20077-s001.docx]

### Supplement 3

### Aerial Imagery Collection and Processing Methods

A brief overview of the aerial imagery collection and processing methods is provided here. See Clarke et al. (2019, 2020) and Willoughby et al. (2021) for comprehensive details. The methods used to estimate transect detection probability from these data are detailed in Ferguson et al. (2023).

During the 2017 and 2022 Eastern Bering Sea (EBS) beluga surveys, data were not collected to estimate the detection probability for beluga groups on the transect line (defined as $\hat{p}_{MR}\left( 0,\boldsymbol{z}_{\boldsymbol{j}}\boldsymbol{;}{\hat{\boldsymbol{\theta}}}_{MR} \right)$ below and in S4). Therefore, we relied on the best information available to us, which was collected during the Aerial Surveys of Marine Mammals (ASAMM) line-transect surveys conducted in the eastern Chukchi and western Beaufort seas from July through October in 2018 and 2019 (Clarke et al. 2019, 2020). We believe the estimates of transect detection probability from ASAMM provide reasonable approximations to the actual value for the 2017 and 2022 EBS beluga aerial surveys based on similarities in survey protocols and beluga group size distributions, which affect detectability. ASAMM surveys targeted belugas and larger cetaceans; marine mammal observer and imagery data were collected concurrently. ASAMM line-transect survey protocols were comparable to those used during the 2017 and 2022 EBS beluga aerial surveys and are detailed in Clarke et al. (2019, 2020). Additionally, the same aircraft (including bubble windows) and marine mammal observer configuration used during the 2017 and 2022 EBS beluga aerial surveys were also used to conduct the ASAMM flights that collected the data we describe below. ASAMM surveys were flown at the same target speed (213 km/h speed) and a similar target altitude (400 m) as the 2017 and 2022 EBS beluga surveys (320 m). Beluga group size distributions were comparable in the 2017 and 2022 EBS beluga data and the relevant ASAMM survey data. For the 2017 EBS beluga survey, 54.7% (338/618) of the sightings were of single belugas, 23.6% (146/618) comprised two belugas, 21.4% (132/618) had 3-10 belugas, and < 1% (2/618) had more than 10 belugas. For the 2022 EBS beluga survey, 75% (180/241) of the sightings were of single belugas, 13% (31/241) comprised two belugas, 8% (19/241) had 3-10 belugas, and 5% (11/241) had more than 10 belugas. In the 2018-2019 ASAMM survey data, 70.9% (720/1015) of the sightings were of single belugas, 17.1% (174/1015) comprised two belugas, 10.6% (108/1015) had 3-10 belugas, and 1.3% (13/1015) had more than 10 belugas.

To estimate transect detection probability for marine mammal observers during ASAMM line-transect surveys, a downward-pointing digital single lens reflex camera with a 20- or 21-mm lens mounted to the belly of the aircraft collected true color (red, green, and blue [RGB]) imagery (Clarke et al. 2019, 2020; Willoughby et al. 2021). At 400 m survey altitude, a single image taken with the 21-mm lens captured a parcel of water measuring approximately 684 m perpendicular to the transect (342 m on each side of the transect) and 457 m along the transect. One image was collected every 2 to 3 seconds, resulting in each parcel of water being visible in three to four images. The imagery served as an “independent observer” for a mark-recapture analysis of the ASAMM aerial observer data.

Willoughby et al. (2021) provide detailed imagery collection and analysis methods and results; here, we present a brief overview. Metadata automatically written to each image included latitude, longitude, date, and time. Every third image collected was manually reviewed post-flight for marine mammal sightings by trained photo analysts. All sightings detected in the imagery were manually compared to the aerial observer database to determine matches based on date, time, and location (side of plane and distance from transect). The results of the matching analysis could be one of three categories: matched, not matched, and “inconclusive results” (abbreviated “IR”). Inconclusive results meant that the photo analyst could not determine for certain whether an imagery sighting was also detected by the aerial observers.

See Ferguson et al. (2023) for comprehensive details on estimating transect detection probability from these data.

Literature Cited

Clarke, J.T., A.A. Brower, M.C. Ferguson, and A.L. Willoughby. 2019. Distribution and relative abundance of marine mammals in the Eastern Chukchi and Western Beaufort Seas, 2018. Annual Report, OCS Study BOEM 2019-021. 451 p.

Clarke, J.T., A.A. Brower, M.C. Ferguson, A.L. Willoughby, and A.D. Rotrock. 2020. Distribution and relative abundance of marine mammals in the Eastern Chukchi Sea, Eastern and Western Beaufort Sea, and Amundsen Gulf, 2019. Annual Report, OCS Study BOEM 2020-027. 603 p.

Ferguson, M.C., A.A. Brower, A.L., Willoughby, and C.K. Sims. 2023. Distribution and estimated abundance of eastern Bering Sea belugas from aerial line-transect surveys in 2017. U.S. Dep. Commer., NOAA Tech. Memo. NMFS-AFSC-471, 50 p.

Willoughby, A., M. Ferguson, B. Hou, C. Accardo, A. Rotrock, A. Brower, J. Clarke, S. Hanlan, M. Foster Doremus, K. Pagan, and L. Barry. 2021. Belly port camera imagery collected to address cetacean perception bias during aerial line-transect surveys: Methods and sighting summaries. U.S. Dep. Commer., NOAA Tech. Memo. NMFS-AFSC-427, 111 p.
