# Supplementary material for: Spatially explicit models of density improve estimates of Eastern Bering Sea beluga (Delphinapterus leucas) abundance and distribution from line-transect surveys
Source: PeerJ. 2025 Sep 30;13:e20077. doi: 10.7717/peerj.20077 (PMC13353160; doi:10.7717/peerj.20077)
Supplement: Supplemental Information 2 [file peerj-13-20077-s002.pdf]

Supplement 1
Glossary of Notation and Abbreviations

a : area searched during line-transect survey, where $a = 2Lw$, L is the total length of transects surveyed, and w is the width of the strip searched on one side of the aircraft

$\mathbf{a}$: vector of the area surveyed for each transect segment ($a_i = 2L_i w$)]

A : total study area size

$\mathbf{A}$: an (n_i, n_η) interpolation matrix used to convert raw random effects into transect-specific values. For SPDE models, this is constructed with a Delauney triangulation. For basis-penalty smooth models, $\mathbf{A}$ is simply the design matrix associated with spatial smooth parameters.

$\mathbf{A}^{pred}$: an (n_h, n_η) interpolation matrix used to convert raw random effects into grid-cell specific predictions. For SPDE models, this is constructed with a Delauney triangulation. For spline-based smoother models, $\mathbf{A}$ is a design matrix associated with locations of grid cell centroids (obtained using the "predict" function in `mgcv`).

$\mathbf{c}$: vector of observed counts of individual animals

c_i : observed number of individual animals on transect segment i

$[\mathbf{c}|\xi, \boldsymbol{\eta}, \mathbf{x}]$: conditional probability density function of observed counts, given parameters, random effects, and known covariates

CV : coefficient of variation

$\hat{D}$: estimate of density of animals (number of animals per unit area)

DSM : density surface model

EBS belugas : Eastern Bering Sea belugas

$g(y_j, \mathbf{z}_j; \hat{\boldsymbol{\theta}}_g)$: probability of detecting an animal at distance y_j, given that it is available to be seen and is associated with covariates $\mathbf{z}_j$, assuming perfect detection on the transect

h : grid cell index

i : segment index

j : group index

k : bootstrap replicate index

L : transect length

n_g : number of groups detected

n_i : number of transect segments in DSM

n_h : number of grid cells in DSM

n_η : number of random effects in DSM

$\hat{N}$: estimate of the total number of animals in the study area

$\hat{p}(\mathbf{z}_j; \hat{\boldsymbol{\theta}})$: model-based estimate of the overall probability that an observer detects a group of whales, given covariates $\mathbf{z}_j$ that affect detectability. This term accounts for all sources of perception and availability bias (Marsh and Sinclair 1989; S4).

$\hat{p}_g(\mathbf{z}_j; \hat{\boldsymbol{\theta}}_g)$: average probability that an observer detects an object that is available to be seen in the area searched, given covariates $\mathbf{z}_j$ that affect detectability, assuming transect detection probability is 1.0

$\mathbf{p}_g$: vector of $\hat{p}_g(\mathbf{z}_j; \hat{\boldsymbol{\theta}}_g)$ for a collection of sightings indexed by j

p_i : shorthand for $\hat{p}(\mathbf{z}_i; \hat{\boldsymbol{\theta}})$ for transects. Note that making the change from group-level detection probability (subscript j) to transect-level detect probability (subscript i) requires that we omit group-specific covariates, such as group size.

$\mathbf{p}_i$: vector of the overall detection probability (including both availability and perception bias corrections) for each segment, p_i.

p_j : shorthand for $\hat{p}(\mathbf{z}_j; \hat{\boldsymbol{\theta}})$ for groups

$p^*(y_j, \mathbf{z}_j; \hat{\boldsymbol{\theta}})$: probability of detecting an animal at distance y_j, given that it is associated with covariates $\mathbf{z}_j$

$\hat{p}_A$: estimate of availability probability, defined as the probability that a group is at the surface within an observer's field of view

$\hat{p}_{MR}(0, \mathbf{z}_j; \hat{\boldsymbol{\theta}}_{MR}$: estimate of transect detection probability, defined as the probability of detecting an animal on the transect (or left-truncation point, if applicable)

$\mathbf{s}$: vector of knot locations for SPDE models

S_j : size of group indexed by j

SPDE : stochasic partial differential equation

$\mathbf{Q}$: precision (inverse covariance) matrix for random effects

Var : variance

w : distance (width) searched on one side of the transect

$\mathbf{x}$: vector of known covariates used in the DSM

y_j : perpendicular distance from the transect line to the sighting of group j

z_i : covariates that affect detectability on segment i

z_j : covariates that affect detectability of group j

β_0 : DSM intercept parameter

$\boldsymbol{\delta}$: vector of 'realized' random effects for transect counts

$\boldsymbol{\eta}$: vector of random effects for the DSM

$[\boldsymbol{\eta}|\mathbf{x}, \boldsymbol{\xi}\ ]$: probability density function of random effects for the DSM

$\hat{\theta}$: parameter estimates required to estimate detection probabilities

μ : mean of a Tweedie probability density function

μ_i : expected number of whales encountered on transect segment i

$\boldsymbol{\xi}$: vector of unknown parameters for the DSM

ρ : power parameter for the Tweedie probability density function

ϕ : dispersion parameter for the Tweedie probability density function

τ : Matérn precision parameter

κ : Matérn inverse range parameter

λ : penalization parameter(s) for basis-penalty smooths

$\lambda_{m,h}$: predicted abundance from model m for unsampled location h

$\lambda_{m,max}$: maximum predicted abundance across all sampled cells
