# Supplementary material for: Spatially explicit models of density improve estimates of Eastern Bering Sea beluga (Delphinapterus leucas) abundance and distribution from line-transect surveys
Source: PeerJ. 2025 Sep 30;13:e20077. doi: 10.7717/peerj.20077 (PMC13353160; doi:10.7717/peerj.20077)
Supplement: Supplemental Information 3 [file peerj-13-20077-s003.docx]

### Supplement 2

### Aerial Line-transect Surveys for Eastern Bering Sea Belugas

### in 2017 and 2022

Survey methods

The Eastern Bering Sea (EBS) beluga study area encompasses Norton Sound and the Yukon River Delta (Figure S2.1). Norton Sound is a shallow bay (average depth 13 m) located along western Alaska, south of the Seward Peninsula, spanning approximately 160 km from Cape Nome to the Yukon River Delta (Figure S2.1). The Sound is seasonally covered with sea ice. During June, sea ice is usually absent, which was the case in June 2017 and 2022. Outflow from the Yukon River creates a nearshore zone of turbid water, extending approximately 40 km offshore, bounded by a sharp oceanographic front, beyond which the waters are more clear and it is possible to see below the surface of the water from an aerial platform.

Aerial line-transect surveys were flown in Norton Sound and along the Yukon River Delta, from 16 to 29 June 2017 (Figure S2.1) and from 24 to 30 June 2022 (Figure S2.2). Following Lowry et al. (2017), systematic transects were placed 9.3 km apart, based on a grid with a randomly selected start point. Transect length varied from approximately 10 to 250 km. Transects were oriented east-west, along lines of latitude, from shore to 166^o^ W. The northernmost transect in both years was located in Norton Bay. The southernmost transect in 2017 was located at 62.3^o^ N, approximately 50 km north of Scammon Bay (Figure S2.1). In 2022, the southern boundary of the study area extended farther south than any previous aerial line-transect survey of EBS belugas had flown (Figures S2.2; Lowry et al. 2017). This southward extension was incorporated into the survey design at the recommendation of the Alaska Beluga Whale Committee to determine the southern extent of the range of EBS belugas. The survey design specified that transects near Scammon Bay would not cross the barrier islands and, therefore, would not enter Scammon Bay (Figure S2.2). This decision follows the convention used during the Aerial Surveys of Arctic Marine Mammals surveys conducted in the eastern Chukchi and western Beaufort seas from 1979-2019 (Clarke et al. 2020). In contrast, at the request of the Alaska Beluga Whale Committee, transects extended into Kokechik Bay and Hooper Bay (Figure S2.2). The total study area was 41,417 km^2^ in 2017 and 47,381 km^2^ in 2022 (Table S2.1).

During the surveys conducted in 2017 and 2022, the Turbo Commander aircraft provided and flown by Clearwater Air, Inc., was based in Nome, Alaska. Unalakleet, Alaska, was an alternate airport that was used for refueling when conducting surveys of the central and southern transects. The Turbo Commander is a twin-turbine, high-wing aircraft. The plane had bubble windows for the left- and right-side primary observers, allowing unobstructed views from directly beneath the plane out to the horizon. Surveys were conducted at 320 m altitude at 213 km/h.

The survey team comprised two primary observers and one dedicated data recorder. The data recorder input sighting data related to detectability and species density into a laptop computer, connected to a GPS, running specialized, menu-driven software (Clarke et al. 2020; MML unpublished report). Time and position data (latitude, longitude, altitude) were automatically recorded in 30-sec intervals or whenever a manual data entry was recorded. Environmental and viewing conditions, including integer-valued Beaufort Sea State, turbidity (binary, yes or no), visibility range perpendicular to the aircraft on each side of the plane (< 1 km, 1-2 km, 2-3 km, 3-5 km, 5-10 km, or unlimited), sky conditions (clear, partly cloudy, overcast), integer-valued sea ice percent (the average from both sides of the plane), and impediments to visibility (glare, fog, haze, precipitation, ice on the window, low ceiling) on each side of the plane were recorded in 5-min intervals or whenever conditions changed.

Primary observers scanned with the naked eye, using binoculars only to check potential targets or get a magnified view on a confirmed target. Declination angles from the horizon to each sighting were measured using handheld clinometers when the sighting was abeam.

One “sighting” or “group” was defined as all animals of the same species within 5 body lengths of each other. Therefore, a group could comprise one or more animals. Belugas in the study area during June are typically distributed in small groups comprising only a few animals. If group size could not be determined with confidence, high and low estimates could also be recorded.

Beluga calves were identified primarily based on size: calves were noticeably smaller than the other animals. In addition to being smaller, coloration (typically grayish or brownish pigmentation) and close proximity to an adult helped observers identify beluga calves. However, it is not always possible for aerial observers to distinguish beluga calves of the year from juveniles; therefore, animals recorded as beluga calves likely include belugas up to a few years old.

Sightings that could not be positively identified to species were recorded at the taxonomic level to which they could be identified (e.g., unidentified cetacean or small unidentified pinniped).

Aerial observers watched for any abrupt and unexpected changes in marine mammals’ initially observed behavior, presumably due to the aircraft. Observed responses and the number of animals that responded were recorded in the database.

Weather permitting, survey effort along each transect was uninterrupted; the aircraft diverted from the transect to circle sightings only in exceptional situations (e.g., to photograph carcasses or investigate sightings of cetaceans that were not belugas, and to confirm species identification). Four survey modes were used for data collection: deadhead, transect, circling from transect, and search. No sighting data were collected during transit or when weather was not conducive to surveying (i.e., during “deadhead” effort). During the remaining three survey modes, observers were actively surveying and all sightings and environmental data were recorded. Transect effort refers to systematic survey effort along a prescribed transect line. Search refers to non-systematic survey effort between transects. Circling from transect occurred when the aircraft diverted from flat and level flight to circle a localized area to investigate a sighting or potential sightings.

Sighting and effort summaries

There was more line-transect survey effort and beluga sightings in 2017 compared to 2022. Ferguson et al. (2023) present detailed results for the 2017 survey; therefore, we provide only an overview here. Between 16 and 29 June 2017, a total of 16 survey flights (62 flight hours) were conducted over 12 days. Each transect in the study area was surveyed at least once, and most transects were surveyed twice (Figure S2.1). The total number of living belugas detected in 2017 was 1,897 (Table S2.1), including 95 calves (as defined above); an additional 2 beluga carcasses were detected. Beluga group sizes during the 2017 surveys ranged from 1 to 39 whales. The geographic stratum with the largest average group size (3.5 belugas per group) was located south of the Yukon Delta (Figure S2.1).

Between 24 and 30 June 2022, a total of 8 survey flights (30 flight hours) were conducted over 4 days. All transects from Pastol Bay to the southern end of the study area (Hooper Bay) were surveyed (Figure S2.2). Most of the transects north of Pastol Bay where relatively high densities of belugas had been detected in previous years (Lowry et al. 2017; Ferguson et al. 2023) were surveyed (Figure S2.2). Due to poor weather, transects in the northern portion of the study area, between Shaktoolik and Stuart Island, were not completed. Figure S2.3 shows all transect effort completed during Beaufort Sea State ≤ 4, color-coded by Beaufort Sea State conditions at the time the survey was conducted. A total of 821 living belugas were detected in 2022 (Table S2.1), including 5 calves; an additional 1 beluga carcass was detected. Beluga group sizes during the 2022 surveys ranged from 1 to 120 whales. The three largest groups were sighted near the barrier islands north of Scammon Bay, an area that had not been surveyed during any previous beluga surveys conducted by the Alaska Beluga Whale Committee or NOAA Fisheries (Lowry et al. 2017; Ferguson et al. 2023; Figure S2.2). The aerial survey observers estimated that these large groups comprised 67, 87, and 120 belugas.

Literature Cited

Clarke, J.T., A.A. Brower, M.C. Ferguson, A.L. Willoughby, and A.D. Rotrock. 2020. Distribution and relative abundance of marine mammals in the Eastern Chukchi Sea, Eastern and Western Beaufort Sea, and Amundsen Gulf, 2019. Annual Report, OCS Study BOEM 2020-027. 603 p.

Ferguson, M.C., A.A. Brower, A.L., Willoughby, and C.K. Sims. 2023. Distribution and estimated abundance of eastern Bering Sea belugas from aerial line-transect surveys in 2017. U.S. Dep. Commer., NOAA Tech. Memo. NMFS-AFSC-471, 50 p.

Lowry, L.F., A. Zerbini, K.J. Frost, D.P. DeMaster, and R.C. Hobbs. 2017. Development of an abundance estimate for the eastern Bering Sea stock of beluga whales (*Delphinapterus leucas*). J. Cetacean Res. Manage. 16: 39-47.

Table S2.1 Summary statistics from the line-transect aerial surveys for belugas conducted in the Norton Sound/Yukon Delta region between 1992 and 2022 by the Alaska Beluga Whale Committee and NOAA Fisheries.

| Survey Dates | Transect Effort (km) | Belugas Counted | Encounter Rate (belugas/km) | Study Area (km^2^) |
| --- | --- | --- | --- | --- |
| 17-21 June 1992 | 7,278 | 1,625 | 0.223 | 6,145 |
| 14-18 June 1993 | 5,539 | 374 | 0.068 | 10,975 |
| 11-16 June 1994 | 5,746 | 370 | 0.064 | 13,965 |
| 5-8 June 1995 | 4,450 | 750 | 0.169 | 19,983 |
| 20-22 June 1995 | 1,776 | 456 | 0.257 | 3,352 |
| 15-17 June 1999 | 3,366 | 589 | 0.175 | 15,794 |
| 17-20 June 2000 | 4,226 | 428 | 0.101 | 38,104 |
| 16-29 June 2017 | 8,587 | 1,897 | 0.221 | 41,416 |
| 24-30 June 2022 | 3,557 | 821 | 0.231 | 47,381 |


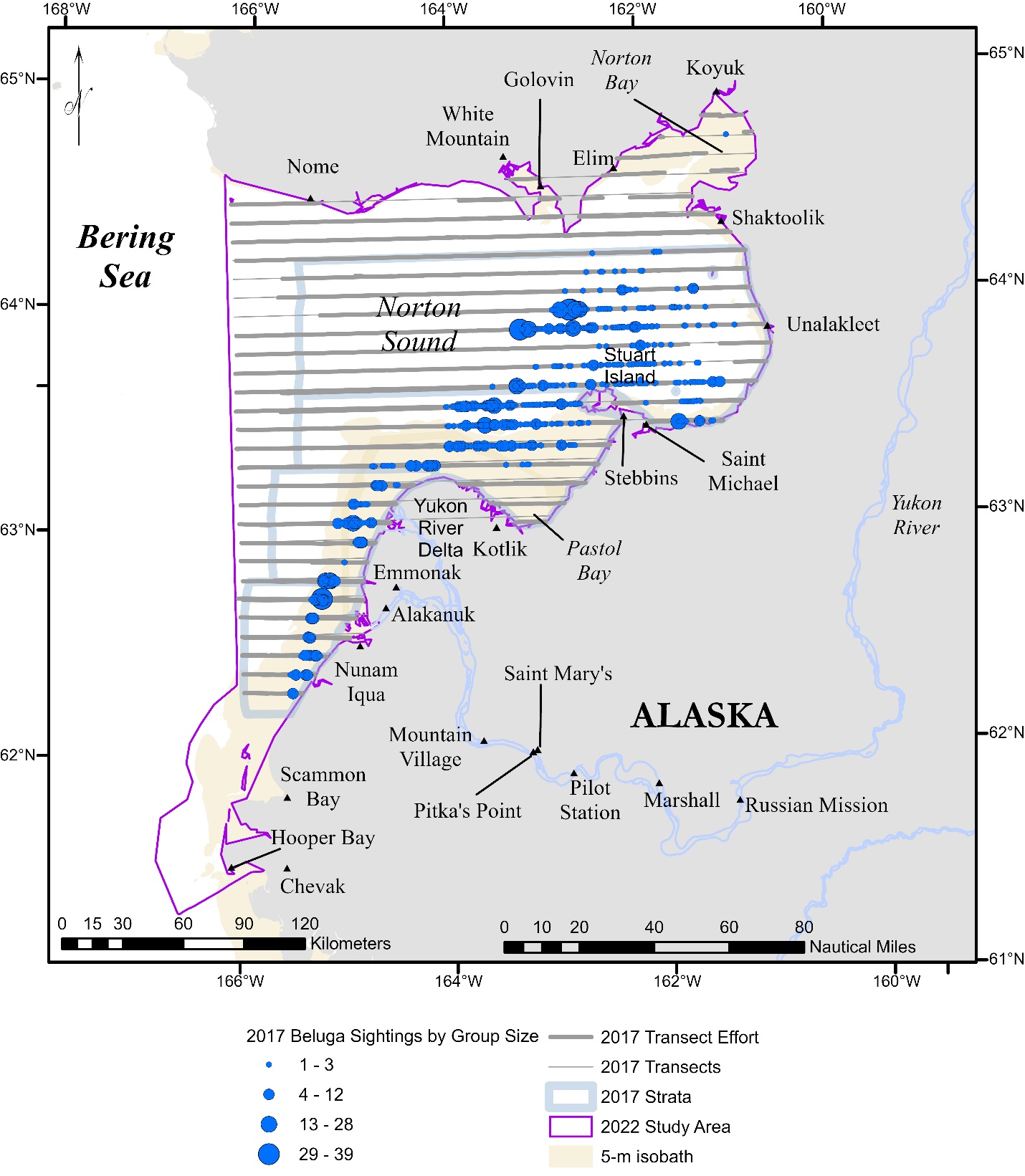


Figure S2.1. 2017 Eastern Bering Sea beluga aerial line-transect survey study area, survey design, and field results. All live beluga sightings and transects flown during Beaufort Sea State ≤ 4 are shown. Waters shallower than 5 m are shaded. The outlines of the geographic strata defined in Lowry et al. (2017) and used in the present analysis are shown. The purple outline represents the extent of the 2022 Eastern Bering Sea study area.


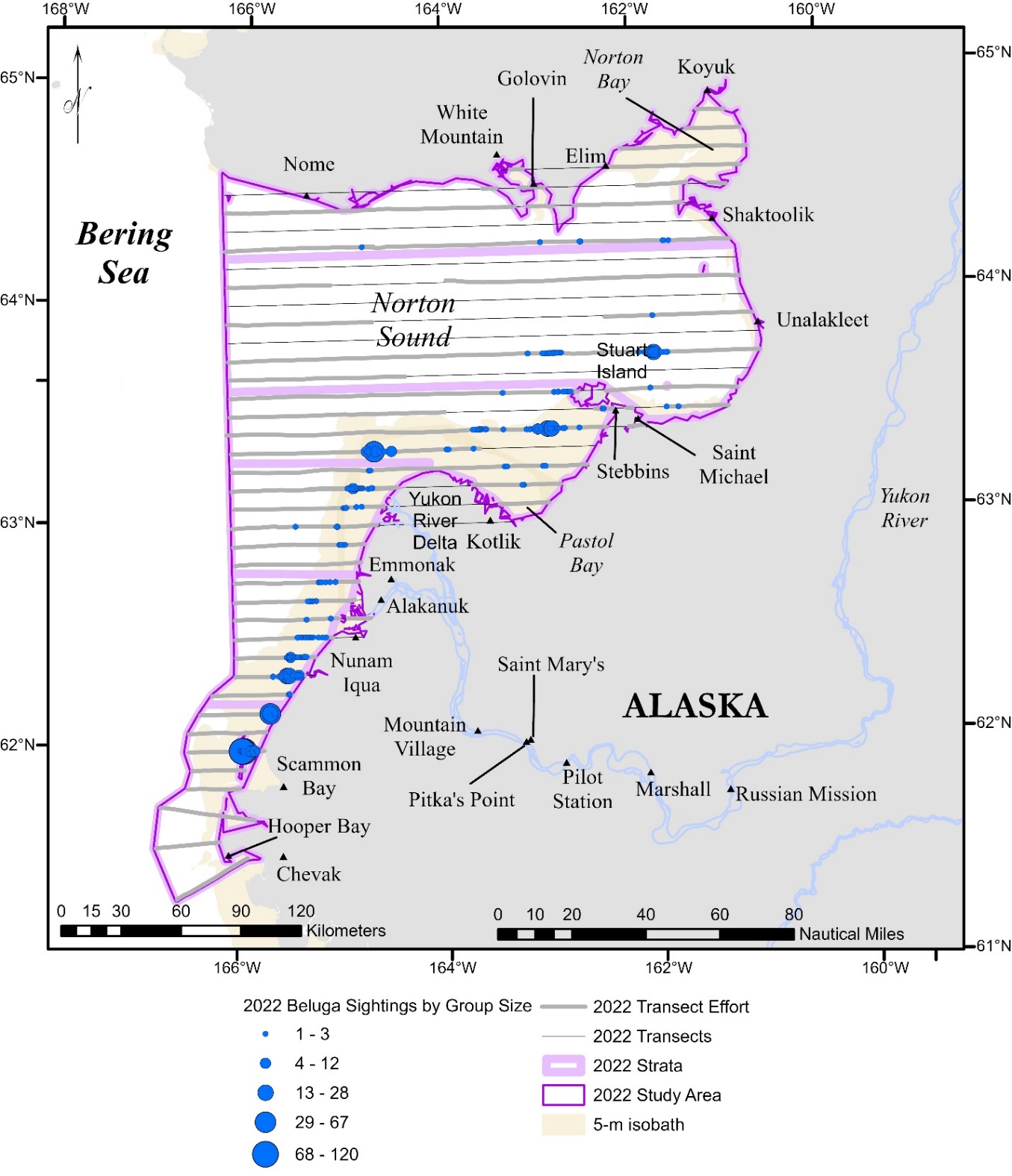


Figure S2.2. 2022 Eastern Bering Sea beluga aerial line-transect survey study area, survey design, and field results. All live beluga sightings and transects flown during Beaufort Sea State ≤ 4 are shown. Waters shallower than 5 m are shaded. Also shown are the outlines of the geographic strata used to derive the abundance estimate from the design-based estimator. Latitudinal boundaries delineating strata are from Lowry et al. (2017). The strata extend to the western border of the 2022 study area. Two new strata were created to encompass the northernmost and southernmost portions of the 2022 study area.


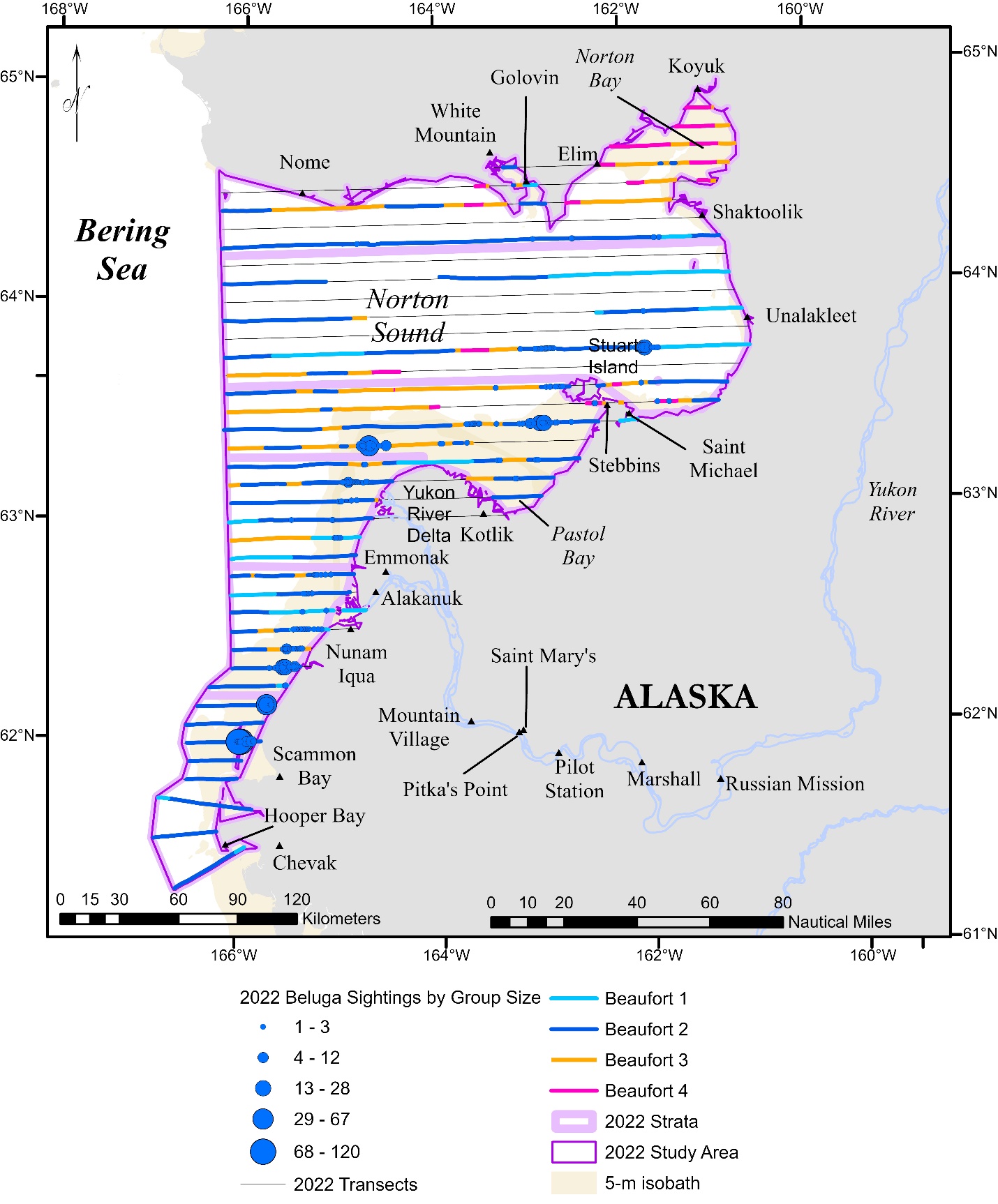


Figure S2.3. 2022 Eastern Bering Sea beluga aerial line-transect survey design. All transects flown during Beaufort Sea State ≤ 4 are shown, color-coded by Beaufort Sea State conditions at the time the survey was conducted.
