# Supplementary material for: Spatially explicit models of density improve estimates of Eastern Bering Sea beluga (Delphinapterus leucas) abundance and distribution from line-transect surveys
Source: PeerJ. 2025 Sep 30;13:e20077. doi: 10.7717/peerj.20077 (PMC13353160; doi:10.7717/peerj.20077)
Supplement: Supplemental Information 4 [file peerj-13-20077-s004.docx]

### Supplement 4

### Eastern Bering Sea Beluga Detection Probabilities

### Basic density estimator

A basic Horvitz-Thompson-like line-transect estimator of animal density is (Buckland et al. 2001; Burt et al. 2014; Marques and Buckland 2003):

|  | $\hat{D}=\frac{1}{a}\sum_{j=1}^{n_{g}} \frac{S_{j}}{\hat{p}\left( \boldsymbol{z}_{j};\hat{\boldsymbol{\theta}} \right)}$ | [1] |
| --- | --- | --- |

where

$n_{g}$ = total number of groups detected;

$S_{j}$ = size of group indexed by *j*;

$a$ = area searched, equal to $2wL$, where $L$ is the total length of transects surveyed and $w$ is the width of the strip searched on one side of the aircraft;

$\hat{p}\left( \boldsymbol{z}_{j};\hat{\boldsymbol{\theta}} \right)$ = estimate of the overall probability that an aerial observer detects group *j* located in the area searched. This probability depends on estimated parameters ($\hat{\boldsymbol{\theta}}$) and other possible covariates ($\boldsymbol{z}_{j}$) that affect detectability. This term accounts for all sources of perception and availability bias (Marsh and Sinclair 1989). Note that $\hat{p}\left( \boldsymbol{z}_{j};\hat{\boldsymbol{\theta}} \right)$ does not depend on distance from the transect line, since we integrate the detection function over the truncation width of the transect as suggested by Marques et al. (2003) to reduce overall variability in the estimator (see Eq. 5 below).

### Eastern Bering Sea beluga observation model

The underlying observation model for the Eastern Bering Sea (EBS) beluga aerial survey was a scaled version of a multiple covariates distance sampling (MCDS) detection function, $g\left( y_{j} ,\boldsymbol{z}_{j}\boldsymbol{;}{\hat{\boldsymbol{\theta}}}_{g} \right)$, (Marques and Buckland 2003, Laake and Borchers 2004):

|  | $\hat{p}^{*}\left( y_{j}, \boldsymbol{z}_{j};\hat{\boldsymbol{\theta}} \right)=\hat{p}_{A}\hat{p}_{MR}\left( 0,\boldsymbol{z}_{j}\boldsymbol{;}{\hat{\boldsymbol{\theta}}}_{MR} \right)g\left( y_{j} ,\boldsymbol{z}_{j}\boldsymbol{;}{\hat{\boldsymbol{\theta}}}_{g} \right)$. | [2] |
| --- | --- | --- |

where

$g\left( y_{j} ,\boldsymbol{z}_{j}\boldsymbol{;}{\hat{\boldsymbol{\theta}}}_{g} \right)$ = probability of detecting an animal at distance $y_{j}$, given that it is available to be seen and is associated with covariates $\boldsymbol{z}_{j}$, assuming perfect detection on the transect;

$\hat{p}^{*}\left( y_{j}, \boldsymbol{z}_{j};\hat{\boldsymbol{\theta}} \right)$ = probability of detecting an animal at distance $y_{j}$, given that it is associated with covariates $\boldsymbol{z}_{j}$. This probability allows for intermittent availability and imperfect detection on the transect.

The MCDS detection function assumes the probability of detecting an object on the transect equals 1.0; it specifies the functional form (shape and scale) of the observation model. The scaling factors in the observation model include an estimated availability probability, $\hat{p}_{A}$, and a mark-recapture component, $\hat{p}_{MR}\left( 0,\boldsymbol{z}_{j}\boldsymbol{;}{\hat{\boldsymbol{\theta}}}_{MR} \right)$.

Availability probability is the probability that a beluga is at the water’s surface and within the observers’ field of view sometime during the period that a plane passes overhead. The mark-recapture component is the probability that a group is detected on the transect line, given that it is at the surface and within the observers’ field of view. Together, $\hat{p}_{A}$ and $\hat{p}_{MR}\left( 0,\boldsymbol{z}_{j}\boldsymbol{;}{\hat{\boldsymbol{\theta}}}_{MR} \right)$ determine the location of the intercept in the observation model.

Values for $\hat{p}_{A}$ and $\hat{p}_{MR}\left( 0,\boldsymbol{z}_{j}\boldsymbol{;}{\hat{\boldsymbol{\theta}}}_{MR} \right)$ were taken from Ferguson et al. (2023) and are described in more detail below. First, we describe how the MCDS detection function was estimated using data from EBS beluga surveys.

### Multiple covariates distance sampling detection function for the EBS beluga aerial surveys

The MCDS detection function was constructed using data only from the 2017 and 2022 EBS beluga aerial surveys, which were filtered prior to fitting the model. Only beluga sightings made by primary observers during transect effort conducted in Beaufort Sea State 0-4 that had recorded declination angles were used to construct the detection function.

Additionally, sighting data were truncated close to and far from the transect. Data were left-truncated to account for lower sighting probabilities very close to the aircraft (Hain et al. 1999). The histogram of perpendicular distances to beluga sightings indicated fewer than expected sightings within 75 m of the transect; therefore, the data were left-truncated at 75 m (Figure S4.1). A total of 887 beluga groups were detected. The farthest 5% of sightings (45 groups) were omitted from the detection function analysis to minimize the effects of outliers. This right-truncation distance was 1.03 km. The width of the strip searched on one side of the aircraft equals the right-truncation distance minus the left-truncation distance, *w* = 0.955 km.

A MCDS model can take various forms, specified by its key function, such as the half-normal key function or hazard-rate key function. For the EBS beluga aerial surveys, MCDS detection function models with half-normal and hazard-rate key functions were considered. A half-normal model in which the standard deviation (scale parameter) is a linear function of covariates affecting detection probability may be represented as:

|  | $g\left( y_{j} ,\boldsymbol{z}_{j}\boldsymbol{;}{\hat{\boldsymbol{\theta}}}_{g} \right)=\exp\left( \frac{-y_{j}^{2}}{2\left[ \exp\left\{ \theta_{0}+\sum_{l} \theta_{j}z_{jl} \right\} \right]^{2}} \right)$ | [3] |
| --- | --- | --- |

An analogous hazard-rate model may be represented as:

|  | $g\left( y_{j} ,\boldsymbol{z}_{j}\boldsymbol{;}{\hat{\boldsymbol{\theta}}}_{g} \right)=1-\exp\left[ -\left( \frac{y_{j}}{\exp\left\{ \theta_{0}+\sum_{l} \theta_{j}z_{jl} \right\}} \right)^{-b} \right]$ | [4] |
| --- | --- | --- |

We used the R package mrds (Laake et al. 2021) to fit MCDS detection functions to observed perpendicular distances of beluga groups, including effects of additional covariates that potentially affect detectability. The covariates that we considered were four different group size variables, turbidity, and Beaufort Sea State (Table S4.1). mrds uses maximum likelihood to fit models. AIC was used to compare the two functional forms for distance data (half-normal vs. hazard-rate), conduct model selection, and ultimately derive maximum likelihood estimates and variances of parameters, ${\hat{\boldsymbol{\theta}}}_{g}$**.** The null hazard-rate models had considerably lower AIC values and exhibited better fit (based on visual inspection of the detection function curve overlaid on the histogram of perpendicular sighting distances) than the half-normal models, so covariate selection proceeded with only the hazard-rate key function. Although four different group size covariates were considered in the initial univariate model, only the covariate with the lowest AIC value among the univariate group size models was retained for further consideration in the model fitting and selection process. The best-fitting MCDS detection function model for EBS belugas included only covariates for Beaufort Sea State and turbidity (Figure S4.2).

The average probability that an aerial observer detects an object that is available to be seen in the area searched, given covariates $\boldsymbol{z}_{j}$ that affect detectability, assuming transect detection probability is 1.0, is (Marques and Buckland 2003):

|  | $\hat{p}_{g}\left( \boldsymbol{z}_{j}\boldsymbol{;}{\hat{\boldsymbol{\theta}}}_{g} \right)=\frac{\int_{0}^{w} g\left( y ,\boldsymbol{z}_{j}\boldsymbol{;}{\hat{\boldsymbol{\theta}}}_{g} \right)dy}{w}$ | [5] |
| --- | --- | --- |

The effective strip half-width (ESW) equals $\hat{p}_{g}\left( \boldsymbol{z}_{j}\boldsymbol{;}{\hat{\boldsymbol{\theta}}}_{g} \right)w$ and it is noteworthy because as many groups are detected within the ESW as are missed beyond the ESW. Due to the left-truncation in the EBS beluga analysis, the ESW stretches from the left-truncation distance to the right-truncation distance.

### Mark-recapture detection probability

The MCDS detection function does not account for animals that are missed on the transect line (or left truncation point, in our case). In absence of a dedicated double-observer study during the EBS beluga surveys, we relied on estimates of ${\hat{\boldsymbol{\theta}}}_{MR}$ from previous surveys of belugas in the Chukchi and Beaufort seas in 2018 and 2019 (Aerial Surveys of Arctic Marine Mammals [ASAMM]; Ferguson et al. 2023) to determine $\hat{p}_{MR}\left( 0,\boldsymbol{z}_{j}\boldsymbol{;}{\hat{\boldsymbol{\theta}}}_{MR} \right)$. Briefly, during ASAMM aerial line-transect surveys, images of whales were collected from a camera system concurrently with visual line-transect survey data. Based on a mark-recapture distance sampling (MRDS) analysis, the proportion of whales detected in the imagery that were also detected by human observers was estimated to be 75.3% ($\hat{p}_{MR}\left( 0,\boldsymbol{z}_{j}\boldsymbol{;}{\hat{\boldsymbol{\theta}}}_{MR} \right)$= 0.753; $\hat{CV}\left[ \hat{p}_{MR}\left( 0,\boldsymbol{z}_{j}\boldsymbol{;}{\hat{\boldsymbol{\theta}}}_{MR} \right) \right]=$ 0.015). Comprehensive details on the MRDS analysis are provided in Ferguson et al. (2023).

### Availability probability

Availability probability, $p_{A}$, is the probability that a group is at the surface within an observer’s field of view (Marsh and Sinclair 1989). Animals that spend a low proportion of their time at the surface where observers can detect them (i.e., low availability) will be detected infrequently relative to their true density (i.e., raw counts will have considerable bias relative to true density). The inverse of availability probability is the availability bias correction factor. Availability probability is a function of the animals’ respiratory patterns and the duration of time in which the ocean at perpendicular distance $y$ is in the observer’s view (i.e., viewing time). We used the estimate of availability probability from Ferguson et al. (2023); therefore, we present only a brief summary of the analytical methods here.

We assumed that the effect of distance on detectability was captured by the MCDS detection function model. Therefore, we used the estimate of availability probability on the transect, $\hat{p}_{A}\left( 0 \right)$, effectively scaling the transect detection probability (Ferguson et al. 2023). Because the field of view from the windows in the EBS beluga survey aircraft was unobstructed ahead of the plane at the left-truncation distance (Ferguson et al. 2021), the length of time for which a sighting was in view on the transect was assumed to be a function of the distance at which a beluga can be detected. The resulting estimate of viewing time on the transect was 15.9 sec.

The best available information on beluga respiration patterns was from behavioral observations made on three adult female belugas tagged with VHF radio tags: one beluga tagged in Bristol Bay, Alaska, in June 1983; and two belugas tagged in Cunningham Inlet, Somerset Island, Canada, in July 1988 (Frost et al. 1985; Frost and Lowry 1995). Using data from these belugas, we determined that, on average, 50% of belugas would be at the surface sometime during the 15.9 sec it took the survey aircraft to pass overhead, resulting in $\hat{p}_{A}=0.5$, equating to an availability bias correction factor of 2.0. There were no estimates of uncertainty for availability probability (Ferguson et al. 2023).

Now, we can decompose the detection probability term $\hat{p}\left( \boldsymbol{z}_{j};\hat{\boldsymbol{\theta}} \right)$ from Eq. 1 into its component parts:

|  | $\hat{p}\left( \boldsymbol{z}_{j};\hat{\boldsymbol{\theta}} \right)={\hat{p}_{A} \hat{p}}_{MR}\left( 0,\boldsymbol{z}_{j}\boldsymbol{;}{\hat{\boldsymbol{\theta}}}_{MR} \right)\hat{p}_{g}\left( \boldsymbol{z}_{j}\boldsymbol{;}{\hat{\boldsymbol{\theta}}}_{g} \right)$. | [6] |
| --- | --- | --- |

### Literature cited

Buckland, S.T., D.R. Anderson, K.P, Burnham, J.L. Laake, D.L. Borchers, and L. Thomas. 2001. *Introduction to Distance Sampling: Estimating Abundance of Biological Populations.* Oxford University Press, Oxford. 432 p.

Burt, M.L., D.L. Borchers, K.J. Jenkins, and T.A. Marques. 2014. Using mark–recapture distance sampling methods on line transect surveys. *Methods in Ecology and Evolution* 5(11): 1180-1191. doi:10.1111/2041-210X.12294.

Ferguson, M.C., A.A. Brower, A.L., Willoughby, and C.K. Sims. 2023. Distribution and estimated abundance of eastern Bering Sea belugas from aerial line-transect surveys in 2017. U.S. Dep. Commer., NOAA Tech. Memo. NMFS-AFSC-471, 50 p.

Ferguson, M.C., J.T. Clarke, A.L. Willoughby, A.A. Brower, and A.D. Rotrock. 2021. Geographically stratified abundance estimate for Bering-Chukchi-Beaufort Seas bowhead whales (*Balaena mysticetus*) from an August 2019 aerial line-transect survey in the Beaufort Sea and Amundsen Gulf. U.S. Dep. Commer., NOAA Tech. Memo. NMFS-AFSC-428, 54 p.

Frost, K. J., and L. F. Lowry. 1995. Radiotag based correction factors for use in beluga whale population estimation. Working paper for Alaska Beluga Whale Committee Scientific Workshop, Anchorage, AK, 5-7 April 1995.

Frost, K. J., L. F. Lowry, and R. R. Nelson. 1985. Radiotagging studies of beluga whales (*Delphinapterus leucas*) in Bristol Bay, Alaska. Mar. Mammal Sci. 1: 191-202.

Hain, J.H.W., S.L. Ellis, R.D. Kenney, and C.K. Slay. 1999. Sightability of right whales in coastal waters of the southeastern United States with implications for the aerial monitoring program, p. 191-208. *In*: G. W. Garner, S. C. Amstrup, J. L. Laake, B. F. J. Manly, L. L. McDonald, and D. G. Robertson (eds.), Marine Mammal Survey and Assessment Methods A. A. Balkema, Rotterdam.

Laake, J.L. and D.L. Borchers. 2004. Methods for incomplete detection at distance zero, p. 109-189. In: S. T. Buckland, D.R. Anderson, K.P. Burnham, J.L. Laake, D.L. Borchers, and L. Thomas (eds.), *Advanced Distance Sampling: Estimating Abundance of Biological Populations*. Oxford University Press, Oxford.

Laake, J., D. Borchers, L. Thomas, D. Miller, and J. Bishop. 2021. mrds: Mark-Recapture Distance Sampling. R package version 2.2.5.9003. Available from: https://github.com/DistanceDevelopment/mrds/.

Marques, F.F.C., and S.T. Buckland. 2003. Incorporating covariates into standard line transect analyses. *Biometrics* 59: 924-935. doi:10.1111/j.0006-341X.2003.00107.x.

Marsh, H., and D. Sinclair. 1989. Correcting for visibility bias in strip transect aerial surveys of aquatic fauna. *Journal of Wildlife Management* 53(4): 1017–1024. doi: 10.2307/3809604.

Table S4.1. Definitions of covariates considered for inclusion in the multiple covariates distance sampling detection function models for the Eastern Bering Sea beluga line-transect aerial surveys in 2017 and 2022.

| Covariate Name | Definition | Categories |
| --- | --- | --- |
| *size* | Observed group size of the sighting |  |
| *loggs* | log_10_(*size*) |  |
| *catsize* | Categorical group size | {1, >1} |
| *catsize3* | Categorical group size | {1, 2, >2} |
| *iBeauf* | Integer-valued Beaufort Sea State |  |
| *Turb* | Turbidity | yes, no |


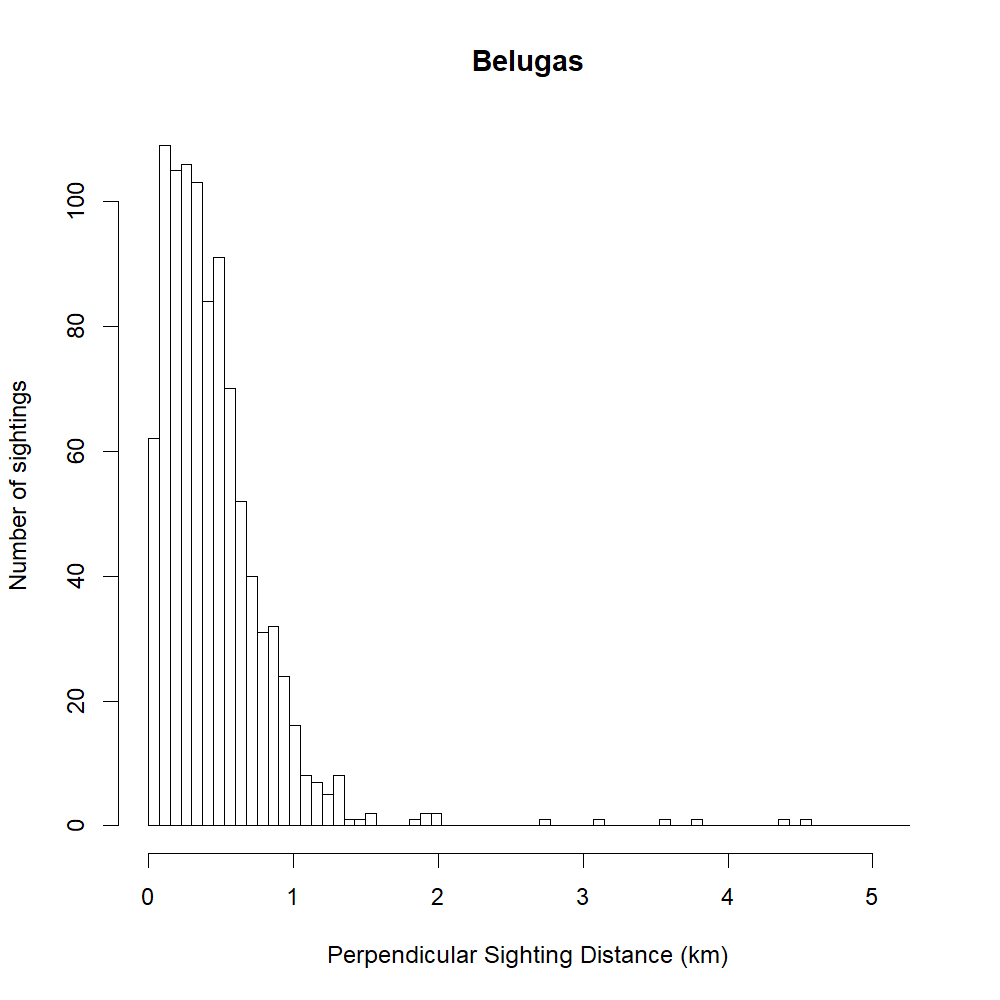


Figure S4.1. Perpendicular distances (km) to all beluga groups detected during the 2017 and 2022 Eastern Bering Sea beluga aerial line-transect surveys. Bin widths are 75 m.


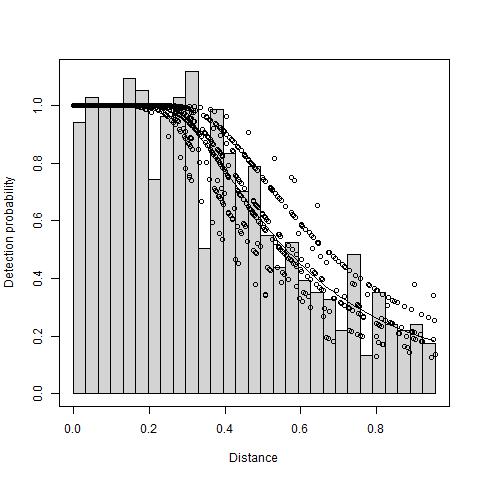


Figure S4.2. Histogram of perpendicular distances to beluga sightings included in the MCDS analysis, with the fit of the selected detection function model overlaid. The selected MCDS model had a hazard-rate key function and scale parameter covariates Beaufort Sea State and Turbidity.
