# Supplementary material for: Spatially explicit models of density improve estimates of Eastern Bering Sea beluga (Delphinapterus leucas) abundance and distribution from line-transect surveys
Source: PeerJ. 2025 Sep 30;13:e20077. doi: 10.7717/peerj.20077 (PMC13353160; doi:10.7717/peerj.20077)
Supplement: Supplemental Information 5 [file peerj-13-20077-s005.docx]

Supplement 5

Bootstrap Pseudo-code for Estimating Uncertainty in

Model-based Estimates of Beluga Abundance

Goal: Incorporate uncertainty from the multiple covariates distance sampling (MCDS) detection function (ddf) model, the spatially-explicit density surface model (DSM), and the mark-recapture distance sampling detection function estimate of transect detection probability into the estimate of uncertainty for the model-based estimated abundance ($\hat{N}$) of belugas from aerial line-transect surveys.

1. Fit MCDS model to EBS beluga sighting data from 2017 and 2022. Obtain: (1) estimated regression parameters, $\hat{\boldsymbol{\beta}}$ and their associated covariance matrix ${\hat{\boldsymbol{\Sigma}}}_{\beta}$; and (2) predictions of detection probability $\tilde{p}_{g,i}$ for each transect segment *i*. In this case, we limit the MCDS coefficients to segment variables, not observation variables. The value $\tilde{p}_{g,i}$ is the average detection probability, computed as the integral of the detection function over the 1/2-width, divided by the 1/2-width (S4 Eq. 5). In S4 Eq. 5, we refer to ${\tilde{\boldsymbol{p}}}_{g}$ using the longhand notation $\hat{p}_{g}\left( \boldsymbol{z}_{\boldsymbol{j}}\boldsymbol{;}{\hat{\boldsymbol{\theta}}}_{g} \right)$. This is the value returned from using the mrds::predict( ) function on a ddf object.
2. Fit each of the *m=1,…,M* DSMs to beluga sighting and effort data in TMB, using the ${\tilde{\boldsymbol{p}}}_{g}$ from step 1 when computing offsets for each count model. The estimated beluga density surfaces from the TMB models are used to compute model-specific estimates of abundance, $\hat{N_{m}}$. We also obtain a conditional variance, $Var(\hat{N_{m}}|{\tilde{\boldsymbol{p}}}_{g})$, from the TMB DSMs. These estimates of $\hat{N_{m}}$ and $Var(\hat{N_{m}}|{\tilde{\boldsymbol{p}}}_{g})$ incorporate a correction for detransformation bias via the epsilon algorithm (Thorson and Kristensen 2016). We are using this approach rather than simulating from the DSMs posterior distributions of random effects because the latter led to problems with extremely high outliers (i.e., implausibly high abundance estimates).
3. To account for increased variance due to uncertainty in ${\tilde{\boldsymbol{p}}}_{g}$, we rely on the law of total variance, which in our context states

$Var\left( \hat{N_{m}} \right)=E(Var\left( {\hat{\boldsymbol{N}}}_{\boldsymbol{m}}\boldsymbol{|}\tilde{p}_{\boldsymbol{g}} \right)\boldsymbol{)+}Var(E(\hat{N_{m}}\boldsymbol{|}{\tilde{\boldsymbol{p}}}_{g}\boldsymbol{)).}$ Eqn 1

For $E(Var\left( \hat{N_{m}} \right|{\tilde{\boldsymbol{p}}}_{g}\boldsymbol{))}$, we simply substitute our conditional variance estimate from the original TMB DSM, $Var(\hat{N_{m}}|{\tilde{\boldsymbol{p}}}_{g})$, obtained in step 2.

Heuristically, the additional piece, $Var(E(\hat{N_{m}}\boldsymbol{|}{\tilde{\boldsymbol{p}}}_{g}\boldsymbol{))}$, describes how abundance estimates might vary with different values of ${\tilde{\boldsymbol{p}}}_{g}$. To estimate this variance, we apply the following bootstrap procedure for each candidate DSM:

- 1. Extract the covariance matrix, ${\hat{\boldsymbol{\Sigma}}}_{\beta}$ for the estimated detection function model (ddf.obj) parameters:

ddf.sigma <- solve(ddf.obj$hessian)

- 1. Extract the detection function model parameters, $\hat{\boldsymbol{\beta}}$:

ddf.beta <- ddf.obj$par

- 1. Implement bootstrap algorithm:

for(k in 1:500){ #repeat the following steps 500 times

- Create bootstrap sample bs.beta for iteration i from the detection function model parameters, assuming a multivariate normal distribution with covariance ddf.sigma:

bs.beta <- mgcv::rmvn(1, ddf.beta, ddf.sigma)

- Generate new bootstrap detection probabilities $\tilde{p}_{g,\left( k \right)}$:
  bs.ddf <- ddf.obj
  bs.ddf$par <- bs.beta
  bs.p <- predict(bs.ddf, gam.data)$fitted
- Re-fit and optimize the TMB DSM for model *m* using the bootstrap detection probabilities bs.p in the calculation of the offset. Compute the bootstrap estimate of abundance for iteration *i*, $\hat{N}_{m,\left( k \right)}$.

} #end bootstrap algorithm

- 1. For bootstrapped DSMs that exhibited numerical convergence, compute $Var\left( \hat{N}_{m,\left( k \right)} \right)$.
  2. Use Eqn 1 to compute $Var\left( \hat{N}_{m} \right)$ from $E(Var\left( \hat{N_{m}} \right|{\tilde{\boldsymbol{p}}}_{g}\boldsymbol{))}$ generated in step 2 and $Var\left( \hat{N}_{m,\left( k \right)} \right)=Var(E(\hat{N_{m}}\boldsymbol{|}{\tilde{\boldsymbol{p}}}_{g}\boldsymbol{))}$ from step 3d. This value $Var\left( \hat{N}_{m} \right)$ is an unconditional estimate of variance that includes uncertainty attributable to MCDS parameters and to the distribution of observed counts.

1. The estimate of abundance for the ensemble model, $\hat{N}_{ens}$, is calculated as the arithmetic average of the $\hat{N}_{m}$ from the candidate DSMs:

$\hat{N}_{ens}=\frac{1}{M}\sum_{m=1}^{M} \hat{N}_{m}$ Eqn 2

1. Calculate the variance of model-averaged predictions using the standard unconditional variance estimator (i.e., Burnham and Anderson 2004, Eq. 4.9):

$\hat{Var}\left( \hat{N}_{ens} \right)=\left[ \sum_{m=1}^{M} w_{m}\sqrt{Var\left( \hat{N}_{m} \right)+\left( \hat{N}_{m}-\hat{N}_{ens} \right)^{2}} \right]^{2}$ Eqn 3

1. Because our estimate of transect detection probability, $p_{MR}\left( 0,\boldsymbol{z}_{\boldsymbol{j}}\boldsymbol{;}{\hat{\boldsymbol{\theta}}}_{MR} \right)$, was derived from aerial line-transect survey data and imagery collected in a different study area during a different year, we assumed that it was independent of the MCDS detection function and the DSMs. Therefore, we used the delta method to incorporate uncertainty from transect detection probability into the estimate of total uncertainty for the ensemble model estimate of abundance:

$CV_{tot}\left( \hat{N}_{ens} \right)=\sqrt{\frac{\hat{Var}\left( \hat{N}_{ens} \right)}{{\hat{N}_{ens}}^{2}}+\left\{ CV\left[ p_{MR}\left( 0,\boldsymbol{z}_{\boldsymbol{j}}\boldsymbol{;}{\hat{\boldsymbol{\theta}}}_{MR} \right) \right] \right\}^{2}}$ Eqn 4

Similarly, the delta function was used to estimate the total CV in estimated abundance from any single DSM:

$CV_{tot}\left( \hat{N}_{m} \right)=\sqrt{\frac{\hat{Var}\left( \hat{N}_{m} \right)}{{\hat{N}_{m}}^{2}}+\left\{ CV\left[ p_{MR}\left( 0,\boldsymbol{z}_{\boldsymbol{j}}\boldsymbol{;}{\hat{\boldsymbol{\theta}}}_{MR} \right) \right] \right\}^{2}}$ Eqn 5

1. There were no estimates of uncertainty for availability probability, $\hat{p}_{A}$ (Ferguson et al. 2013); therefore, this parameter did not contribute to the estimated uncertainty in abundance.
