# Supplementary material for: Spatially explicit models of density improve estimates of Eastern Bering Sea beluga (Delphinapterus leucas) abundance and distribution from line-transect surveys
Source: PeerJ. 2025 Sep 30;13:e20077. doi: 10.7717/peerj.20077 (PMC13353160; doi:10.7717/peerj.20077)

Supplement 6
DSM Overview and Evaluation for the Eastern Bering Sea Beluga Case Study

Table S6.1. Key aspects of all 2017 Eastern Bering Sea beluga density surface models constructed and evaluated. Blue highlighting indicates models included in the ensemble.

Year	Spatial Formulation	Number of Observations	Number of Transect Segments	Number of Prediction Cells	Number of Nodes	max.edge Inside (Outside)	Cutoff	Inner Boundary	Outer Boundary	Smoothing Spline Variables	number unsampled cells with extreme predicted abundance
2017	SPDE Matérn	598	604	342	595	25 (50)	0.2*max.edge	non-convex hull around all segment midpoints, plus a buffer of 25 km	offset from the inner boundary by a distance equal to 1/3 the latitudinal range of the study area	NA	0
2017	SPDE Matérn	598	604	342	564	25 (50)	0.2*max.edge	non-convex hull around all segment midpoints	offset from the inner boundary by a distance equal to 35% of the inner boundary diameter	NA	0
2017	SPDE Matérn	598	604	342	528	25 (50)	0.2*max.edge	non-convex hull around all segment midpoints	offset from the inner boundary by a distance equal to 20% of the inner boundary diameter	NA	0
2017	SPDE Matérn	598	604	342	289	50 (100)	0.2*max.edge	non-convex hull around all segment midpoints	offset from the inner boundary by a distance equal to 35% of the inner boundary diameter	NA	0
2017	SPDE Matérn	598	604	342	160	75 (150)	0.2*max.edge	non-convex hull around all segment midpoints	offset from the inner boundary by a distance equal to 35% of the inner boundary diameter	NA	0
2017	SPDE Matérn	598	604	342	199	60 (120)	0.2*max.edge	non-convex hull around all segment midpoints	offset from the inner boundary by a distance equal to 35% of the inner boundary diameter	NA	0
2017	SPDE Matérn with barriers	598	604	342	660	25 (50)	0.2*max.edge	coastline and study area boundaries	NA	NA	0
2017	SPDE Matérn with barriers	598	604	342	292	50 (100)	0.2*max.edge	coastline and study area boundaries	NA	NA	0
2017	SPDE Matérn with barriers	598	604	342	175	75 (150)	0.2*max.edge	coastline and study area boundaries	NA	NA	0
2017	SPDE Matérn with barriers	598	604	342	214	60 (120)	0.2*max.edge	coastline and study area boundaries	NA	NA	0
2017	Soap film smoother	598	604	342	NA	NA	NA	NA	NA	s(easting, northing, bs="so", k=150)	0
2017	Tensor product smoother	598	604	342	NA	NA	NA	NA	NA	te(easting, northing, bs="ts", k=14)	0
2017	Bivariate isotropic tprs	598	604	342	NA	NA	NA	NA	NA	s(easting, northing, bs="ts", k=200)	0

Table S6.2. Key aspects of all 2022 Eastern Bering Sea beluga density surface models constructed and evaluated. Blue highlighting indicates models included in the ensemble.

Year	Spatial Formulation	Number of Observations	Number of Transect Segments	Number of Prediction Cells	Number of Nodes	max.edge Inside (Outside)	Cutoff	Inner Boundary	Outer Boundary	Smoothing Spline Variables	number unsampled cells with extreme predicted abundance
2022	SPDE Matérn	241	317	554	831	25 (50)	0.2*max.edge	non-convex hull around all segment midpoints	offset from the inner boundary by a distance equal to 35% of the inner boundary diameter	NA	0
2022	SPDE Matérn	241	317	554	257	75 (150)	0.2*max.edge	non-convex hull around all segment midpoints	offset from the inner boundary by a distance equal to 35% of the inner boundary diameter	NA	0
2022	SPDE Matérn	241	317	554	351	50 (100)	0.2*max.edge	non-convex hull around all segment midpoints	offset from the inner boundary by a distance equal to 35% of the inner boundary diameter	NA	0
2022	SPDE Matérn	241	317	554	308	60 (120)	0.2*max.edge	non-convex hull around all segment midpoints	offset from the inner boundary by a distance equal to 35% of the inner boundary diameter	NA	0
2022	SPDE Matérn	241	317	554	326	55 (110)	0.2*max.edge	non-convex hull around all segment midpoints	offset from the inner boundary by a distance equal to 35% of the inner boundary diameter	NA	0
2022	SPDE Matérn	241	317	550	308	60 (120)	0.2*max.edge	non-convex hull around all segment midpoints; omit Scammon	offset from the inner boundary by a distance equal to 35% of the inner boundary diameter	NA	0
2022	SPDE Matérn with barriers	241	317	550	953	25 (50)	0.2*max.edge	coastline and study area boundaries; omit Scammon	NA	NA	0
2022	SPDE Matérn with barriers	241	317	550	350	55 (110)	0.2*max.edge	coastline and study area boundaries; omit Scammon	NA	NA	0
2022	SPDE Matérn with barriers	241	317	550	316	60 (120)	0.2*max.edge	coastline and study area boundaries; omit Scammon	NA	NA	0
2022	SPDE Matérn with barriers	241	317	550	258	75 (150)	0.2*max.edge	coastline and study area boundaries; omit Scammon	NA	NA	0
2022	SPDE Matérn with barriers	241	317	550	782	30 (60)	0.2*max.edge	coastline and study area boundaries; omit Scammon	NA	NA	1
2022	SPDE Matérn with barriers	241	317	550	616	40 (80)	0.2*max.edge	coastline and study area boundaries; omit Scammon	NA	NA	1
2022	SPDE Matérn with barriers	241	317	550	702	35 (70)	0.2*max.edge	coastline and study area boundaries; omit Scammon	NA	NA	1
2022	SPDE Matérn with barriers	241	317	550	387	50 (100)	0.2*max.edge	coastline and study area boundaries; omit Scammon	NA	NA	0
2022	Soap film smoother	241	317	554	NA	NA	NA	NA	NA	s(easting, northing, bs="so", k=222)	0
2022	Tensor product smoother	241	317	554	NA	NA	NA	NA	NA	te(easting, northing, bs="ts", k=18)	0
2022	Bivariate isotropic tprs	241	317	554	NA	NA	NA	NA	NA	s(easting, northing, bs="ts", k=309)	0

2017 candidate models in ensemble

2017 TMB SPDE mesh6 (max.edge = 60 km)

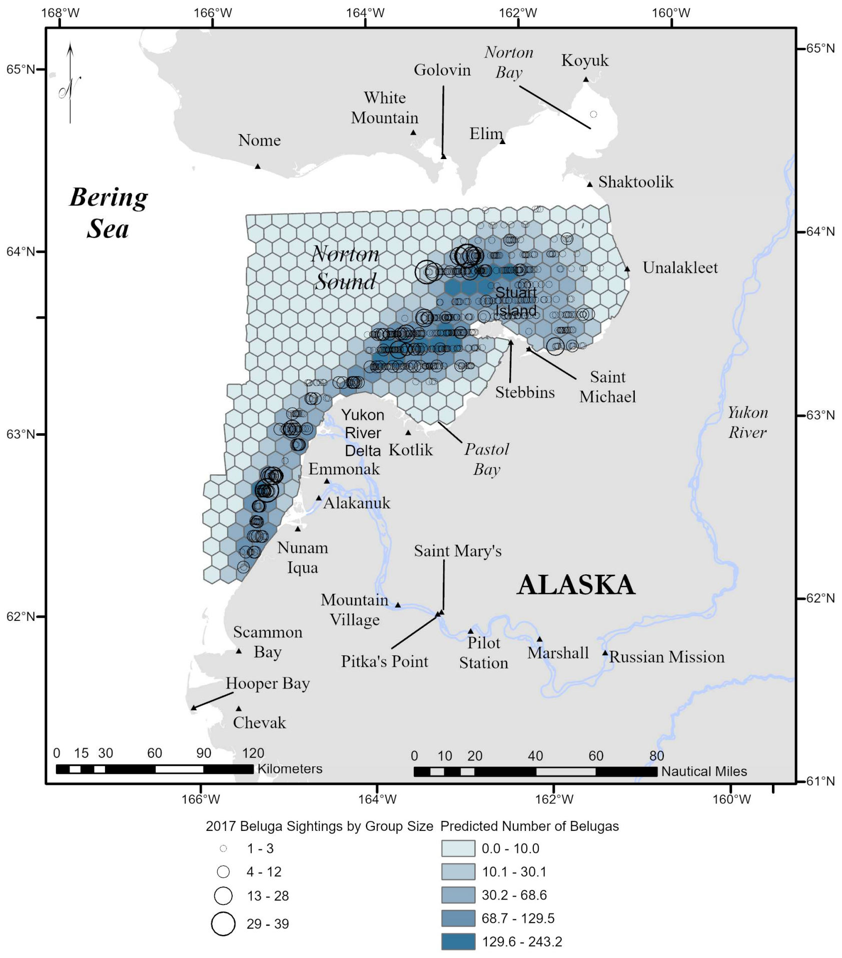

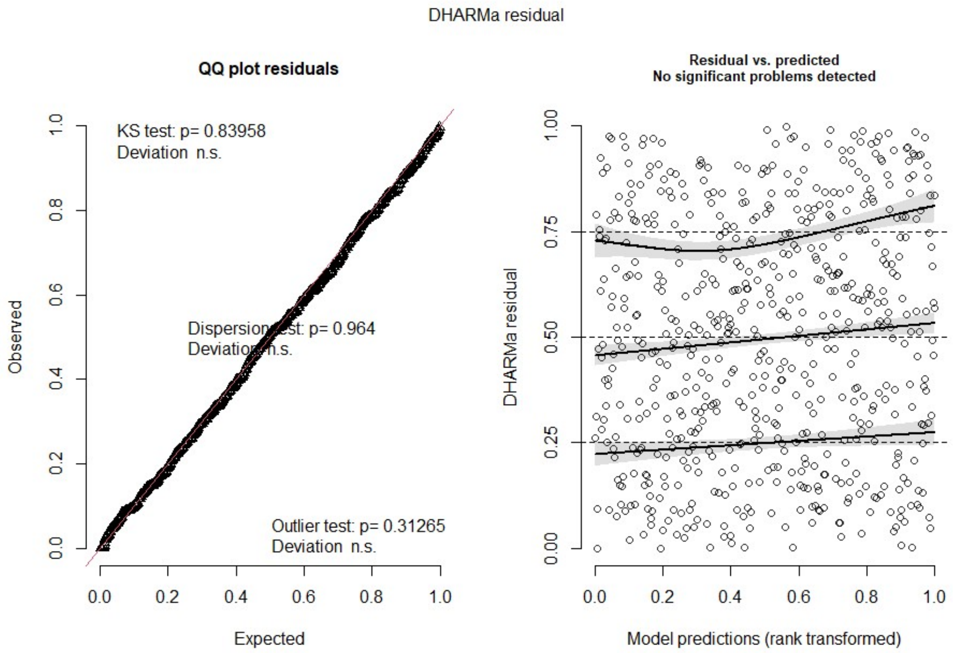

2017 TMB SPDE mesh6 Variance/Mean

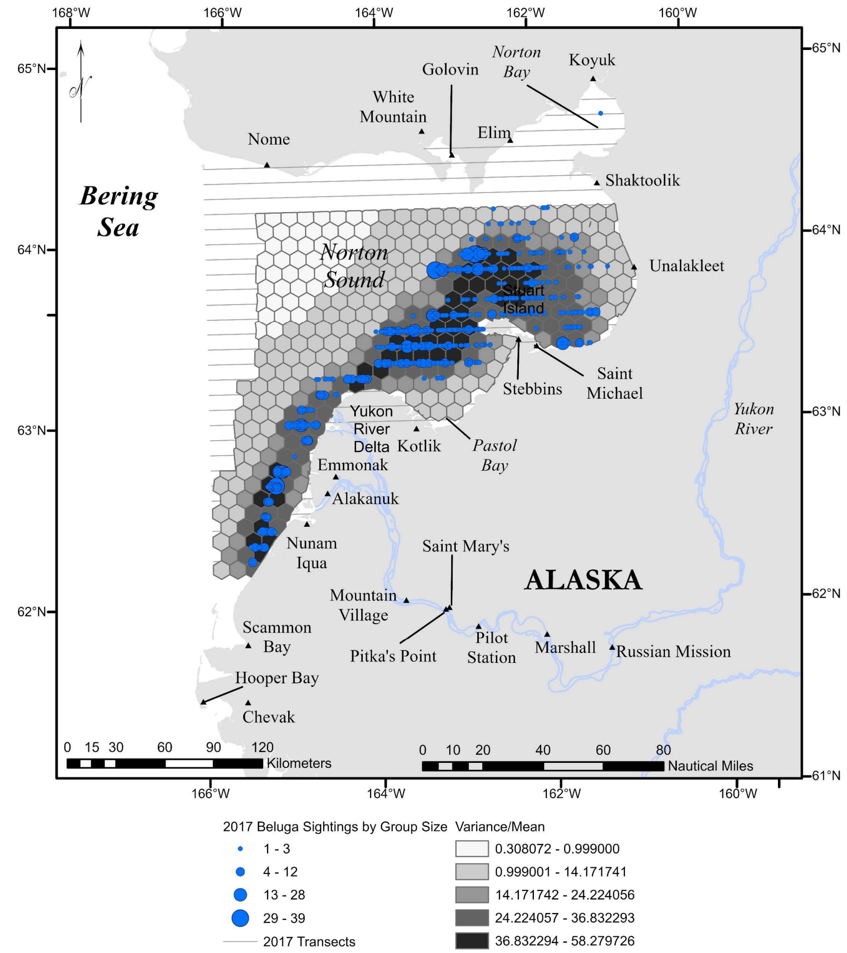

2017 TMB soap(easting,northing)

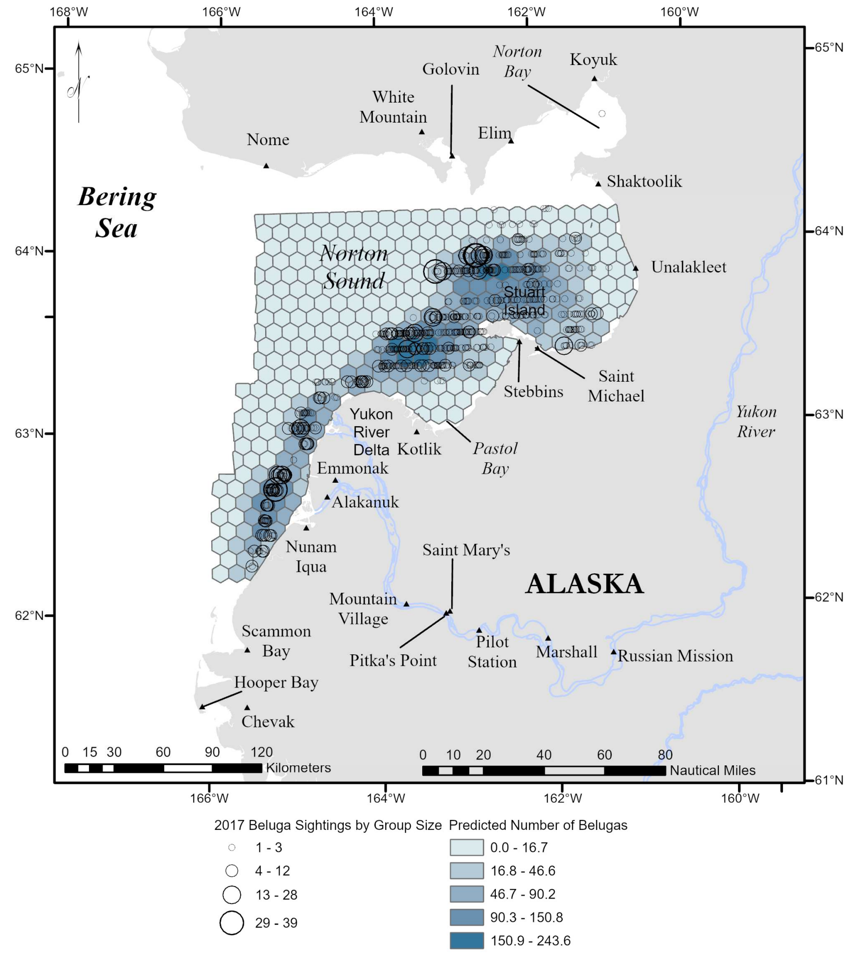

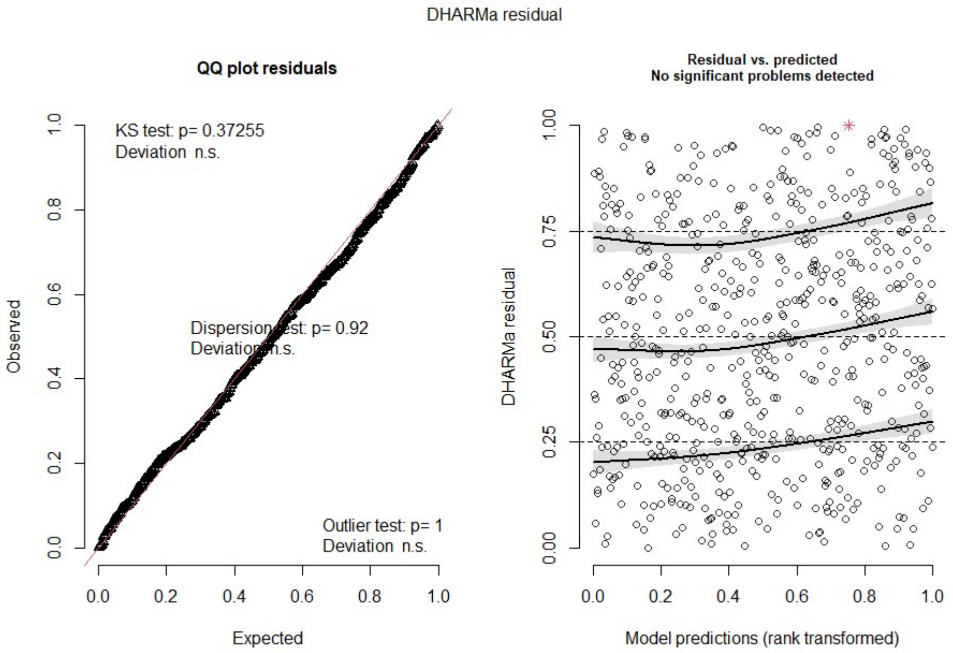

2017 TMB te(easting,northing)

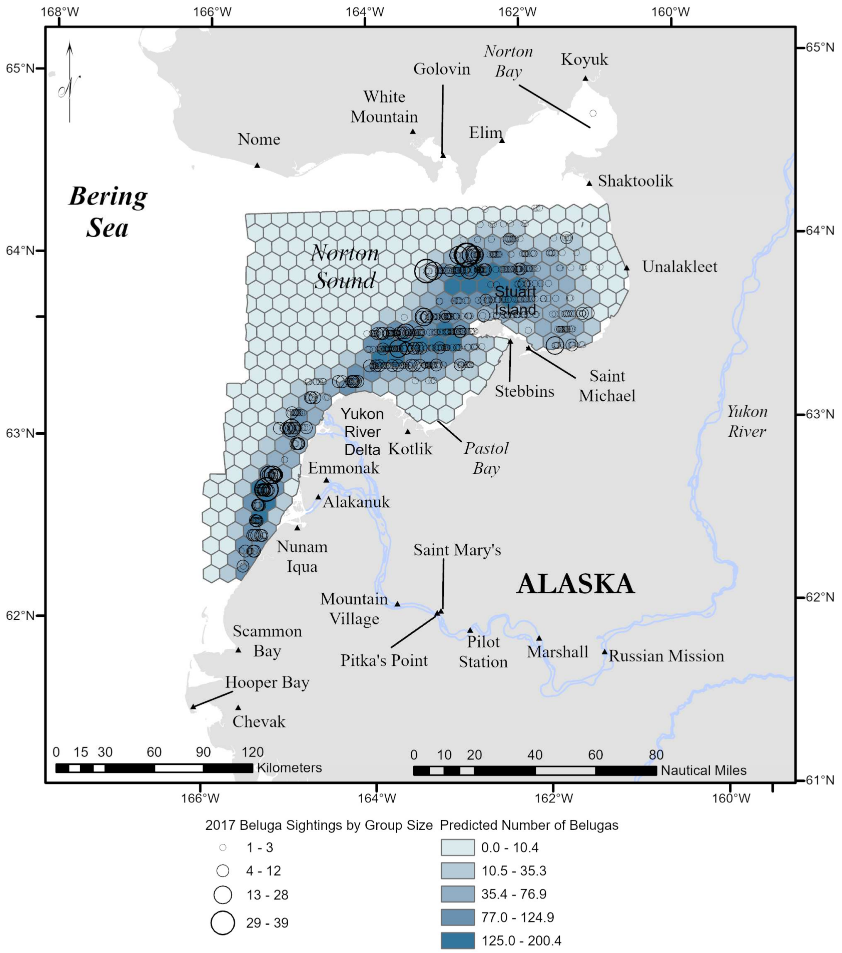

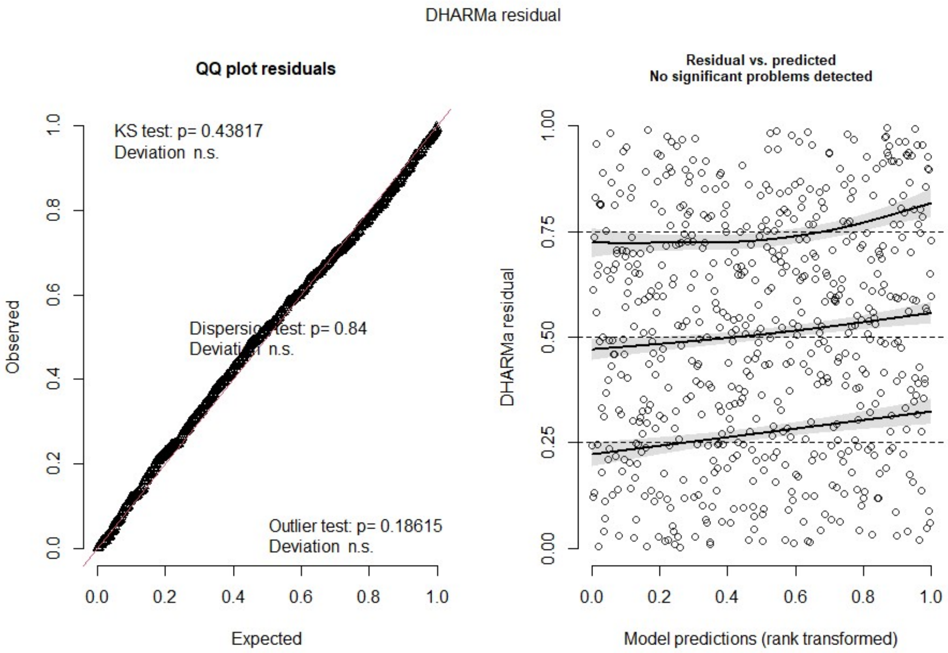

2017 TMB s(easting,northing,bs=“ts”)

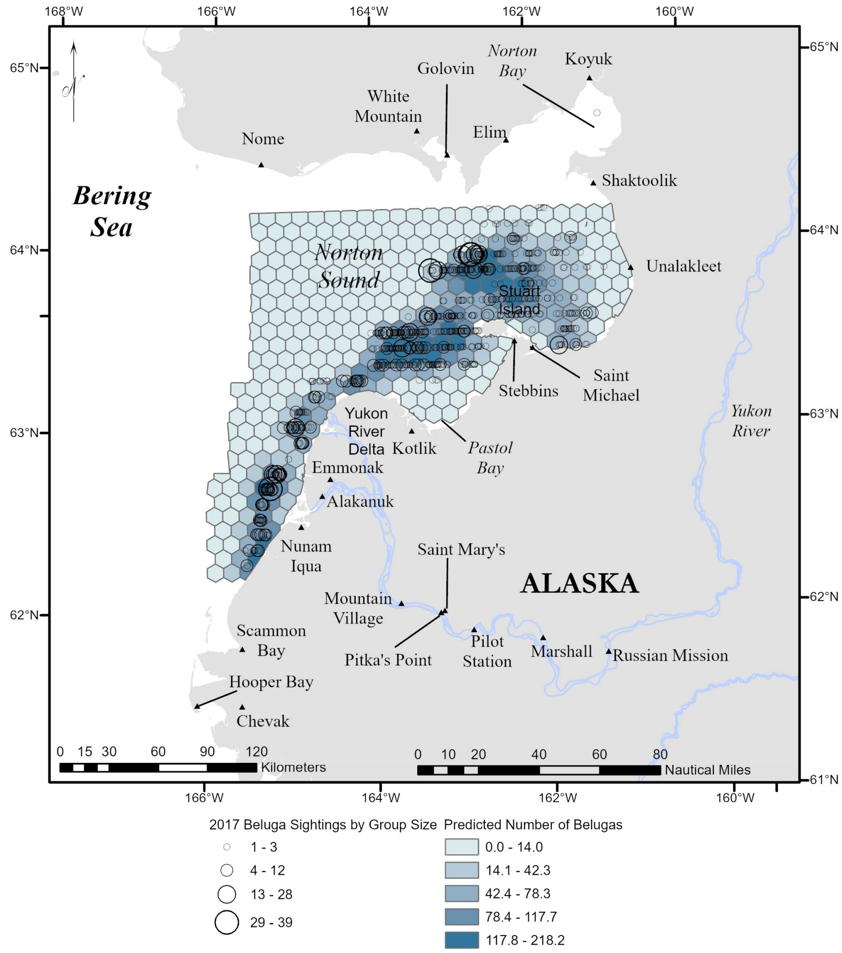

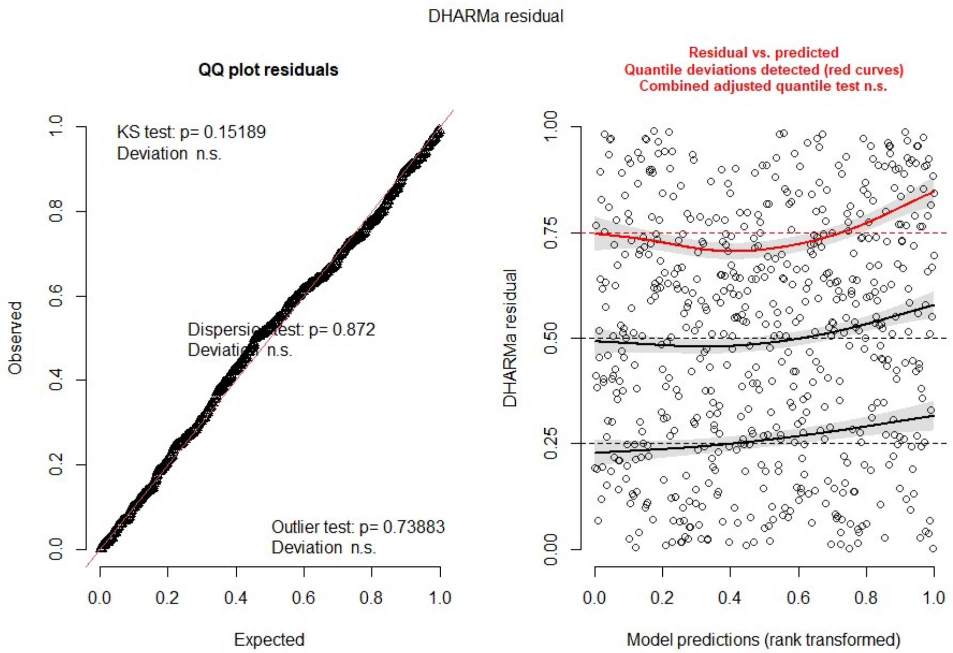

2022 candidate models in ensemble

2022 TMB SPDE no Scammon mesh5 (max.edge = 60 km)

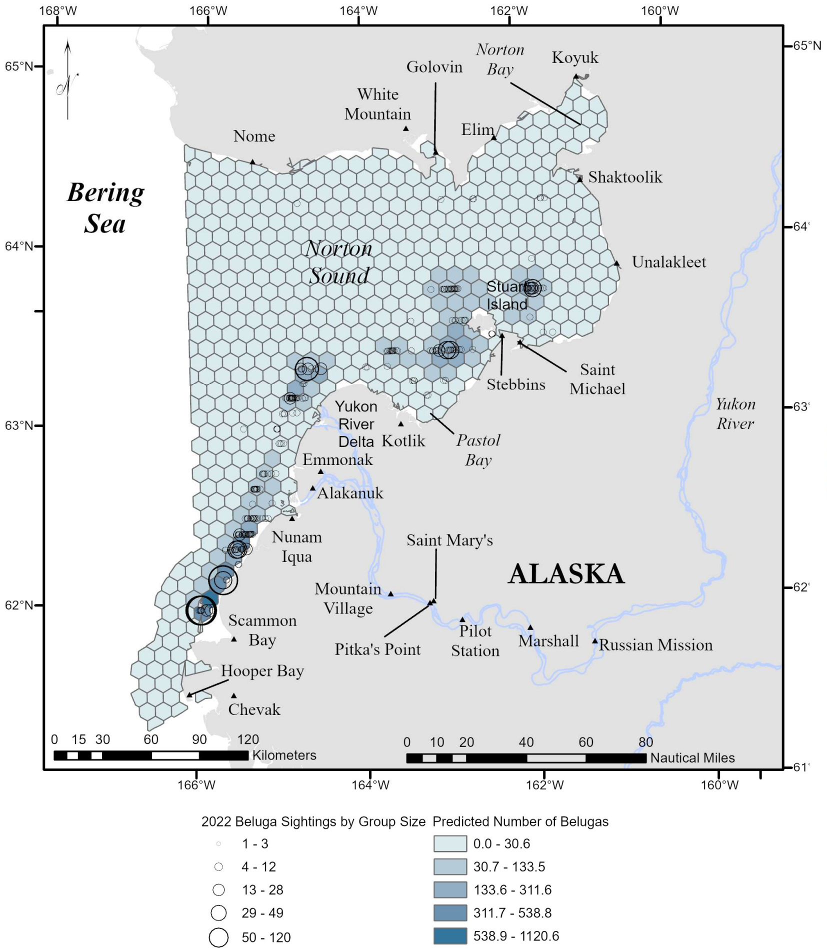

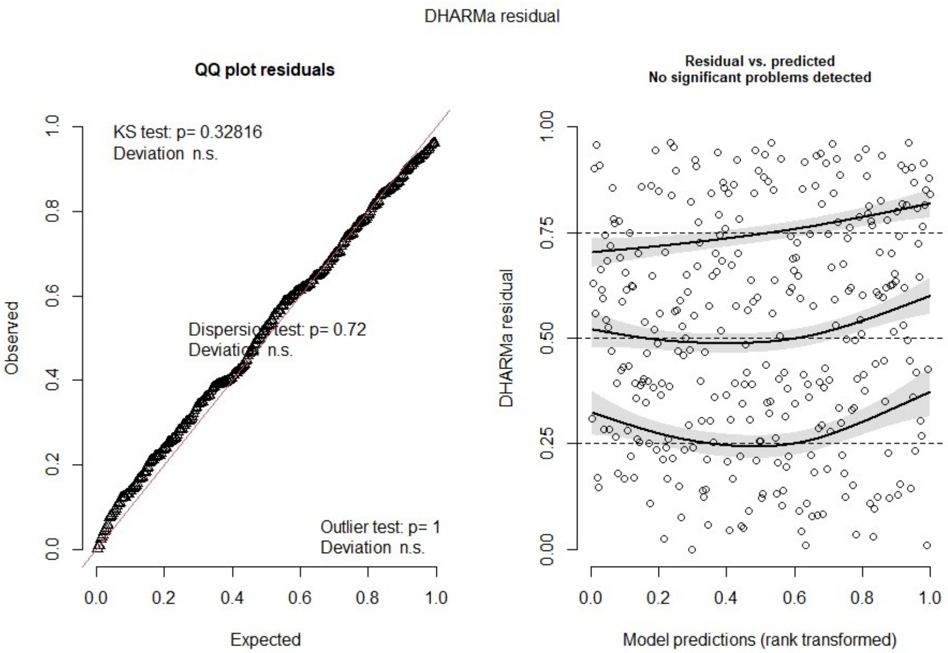

2022 TMB SPDE w/barriers no Scammon mesh2 (max.edge = 60 km)

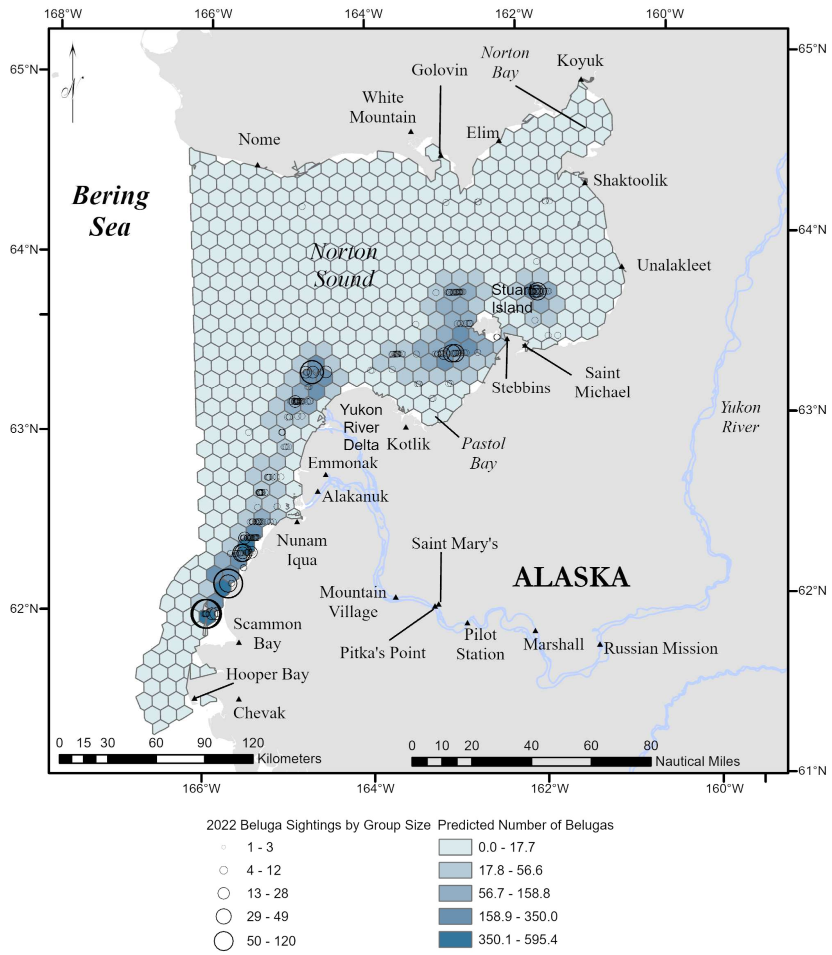

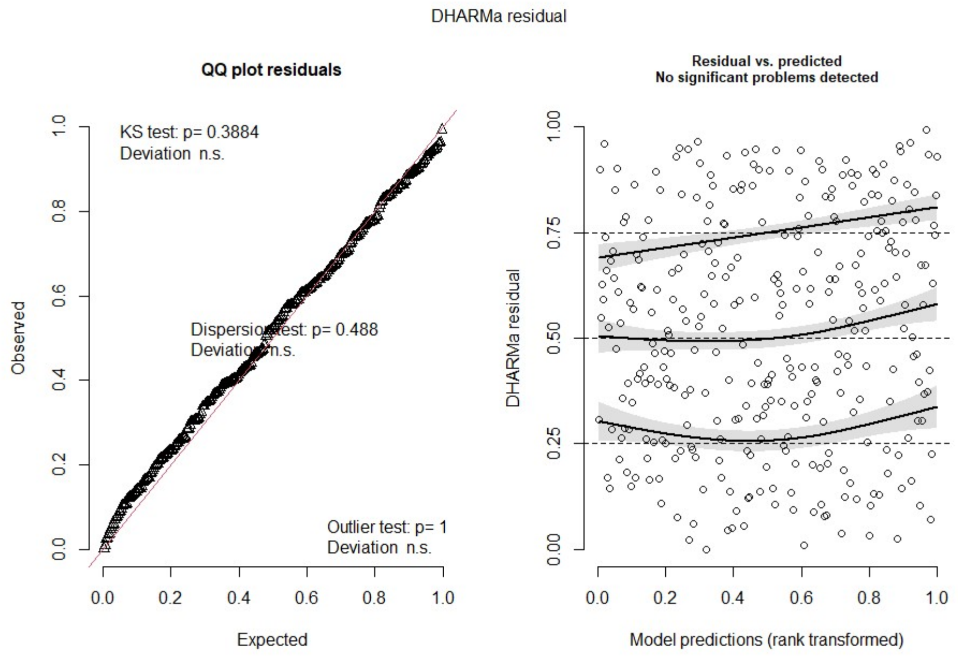

2022 TMB soap(easting,northing)

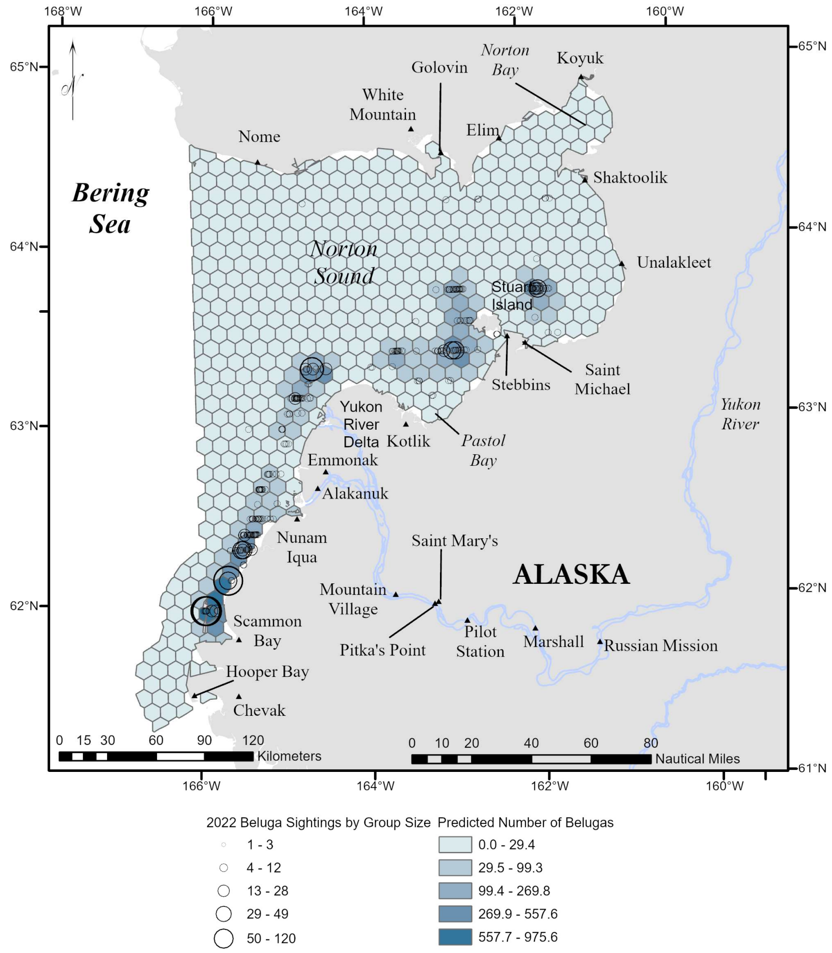

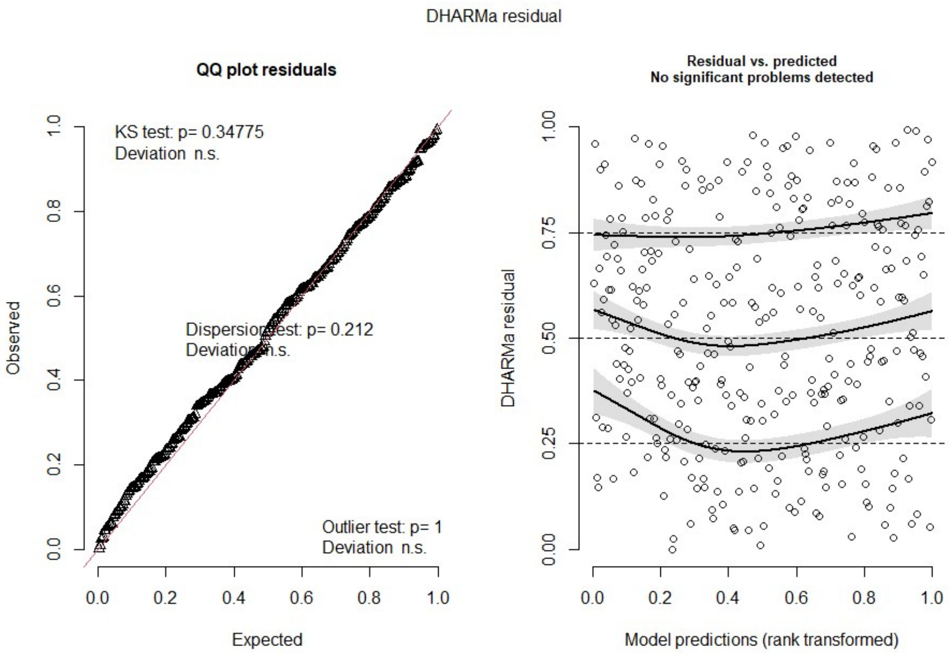

Supplement: Supplemental Information 6 [file peerj-13-20077-s006.pdf]
